# Adsorbent based on MOF-5/cellulose aerogel composite for adsorption of organic dyes from wastewater

**DOI:** 10.1038/s41598-024-65774-y

**Published:** 2024-07-07

**Authors:** Mohammad Shiri, Majid Hosseinzadeh, Soudeh Shiri, Shahrzad Javanshir

**Affiliations:** 1 School of Civil Engineering, Iran University of Science and Technology, Narmak, Tehran, Iran; 2 Department of Organic Colorants, Institute of Color Science and Technology, Tehran, Iran; 3 Pharmaceutical and Heterocyclic Chemistry Research Laboratory, Department of Chemistry, Iran University of Science and Technology, Tehran, 16846-13114 Iran

**Keywords:** MOF-5/cellulose aerogel composite, Pampas, Wastewater treatments, Adsorption capacity, Acid blue dye, Environmental chemistry, Environmental sciences, Chemistry

## Abstract

Industries persistently contribute to environmental pollution by releasing a multitude of harmful substances, including organic dyes, which represent a significant hazard to human health. As a result, the demand for effective adsorbents in wastewater treatment technology is steadily increasing so as to mitigate or eradicate these environmental risks. In response to this challenge, we have developed an advanced composite known as MOF-5/Cellulose aerogel, utilizing the Pampas plant as a natural material in the production of cellulose aerogel. Our investigation focused on analyzing the adsorption and flexibility characteristics of this novel composite for organic dye removal. Additionally, we conducted tests to assess the aerogel’s reusability and determined that its absorption rate remained consistent, with the adsorption capacity of the MOF-5/cellulose aerogel composite only experiencing a marginal 5% reduction. Characterization of the material was conducted through XRD analysis, revealing the cubic structure of MOF aerogel particles under scanning electron microscopy. Our study unequivocally demonstrates the superior adsorption capabilities of the MOF-5/cellulose aerogel composite, particularly evident in its efficient removal of acid blue dye, as evaluated meticulously using UV–Vis spectrophotometric techniques. Notably, our findings revealed an impressive 96% absorption rate for the anionic dye under acidic pH conditions. Furthermore, the synthesized MOF-5/cellulose aerogel composite exhibited Langmuir isotherm behavior and followed pseudo-second-order kinetics during the absorption process. With its remarkable absorption efficiency, MOF-5/cellulose aerogel composites are poised to emerge as leading adsorbents for water purification and various other applications.

## Introduction

Water is vital for all forms of life, encompassing humans, animals, and plants. Despite its critical role, escalating pollution levels and water scarcity have emerged as significant global environmental challenges, erecting substantial barriers. Industrial activities, daily human practices, and agricultural operations stand out as prominent contributors to water pollution. Particularly, industrial wastewater contains perilous substances that pose significant threats to human health and aquatic ecosystems. Urgent action is imperative to address this issue before irreparable damage occurs^1,2^.Various chemical and physical techniques, including adsorption, chemical oxidation and reduction, biological treatment, and photocatalytic degradation, have been employed for wastewater treatment^3^. Among these methodologies, adsorption technology has gained prominence due to its effectiveness, cost-efficiency, and ease of application, employing a diverse array of adsorbent materials. There remains a persistent interest in refining adsorption capacity and conditions, whether through the exploration of innovative adsorbents or the modification of existing ones^4,5^. The ideal adsorbent candidate would possess high capacity, water stability, reusability, and affordability^6–8^. Metal–organic frameworks (MOFs) demonstrate remarkable adsorption properties and can be tailored to target specific dye molecules, rendering them highly advantageous for wastewater treatment.

However, the presence of metal groups or ions and ligand groups in the MOF structure facilitates strong interactions between the dye molecules and the MOF, resulting in enhanced absorption. These interactions primarily occur through electrostatic forces between species with differing surface charges. Different dyes can engage with MOFs via electrostatic interactions^9,10^. Dyes containing aromatic rings in their structure are removed through π–π interactions with aromatic MOFs. Additionally, hydrogen bonds formed between dyes and MOFs play a crucial role in the removal process. Hydrogen bonds form when hydrogen atoms bond with electronegative atoms. If the MOF structure contains two OH and N groups, a hydrogen bond can form between the dye and the MOF^11,12^. These diverse interactions collectively contribute to the overall adsorption process, enabling MOFs to effectively remove dyes from wastewater. By customizing the structure and functional groups of MOFs, their adsorption capacity and selectivity for specific dyes can be further enhanced. Despite their effectiveness in adsorption, MOFs are susceptible to decomposition when exposed to hydrophilic media. To address this vulnerability and improve their stability, MOFs are often combined with suitable materials to form new composites with enhanced properties and water stability^13^. The formation of MOF-based composites involves integrating MOFs into supporting materials or matrices, providing structural reinforcement and safeguarding the MOF structure from degradation in aqueous environments.

Cellulose aerogel represents an ultra-light porous material with a three-dimensional network structure^14–16^. It offers advantages such as low density, high porosity, recyclability, biodegradability, and low cost, along with a high specific surface area, rendering it an excellent candidate as a supporting material. Furthermore, cellulose aerogel demonstrates both physical and chemical stability in water and can be easily retrieved from aqueous environments^17–23^ As a novel type of MOF, carbon materials derived from ZIF-8 possess distinctive characteristics, including an elevated specific surface area and stratified porous arrangement. These features endow them with abundant accessible active sites and reduce the distance for reactants to diffuse^24^. Zhu et al. successfully combined various MOF materials (ZIF-8, UiO-66, and MIL-100(Fe)) with cellulose aerogel for the efficient adsorption of heavy metals. Wang et al. loaded UiO-66 onto cellulose aerogel to facilitate the adsorption of dyes^25^. Similarly, Ma et al. employed an in-situ growth method to prepare ZIF-8@cellulose aerogel, achieving effective adsorption of both dyes and heavy metal ions. However, MOF materials are primarily found in powder form, which often leads to agglomeration, making them challenging to recycle. Additionally, some MOF materials exhibit poor stability in water, limiting their applications in water treatment. Therefore, immobilizing MOFs onto a suitable support material is an effective strategy for facilitating their recovery and reuse^26^.

**Figure 1:**
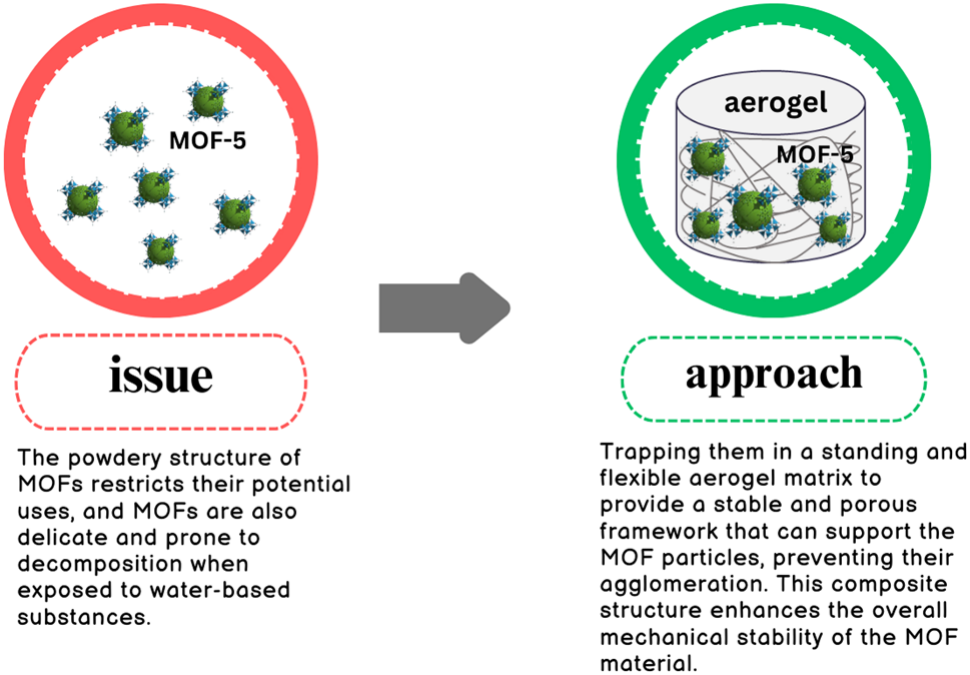
Performance compression of MOF and MOF/cellulose aerogel composite.

Furthermore, pampas grass emerges as a valuable resource for composite production. As a naturally occurring plant, it serves as an excellent source for creating cellulose aerogel. Cellulose aerogels derived from pampas grass possess unique qualities such as biodegradability, biocompatibility, renewability, and minimal toxicity. These inherent properties confer significant advantages to cellulose aerogels^27^. Figure 1 compares the performance of MOF and MOF with cellulose aerogel. The effective utilization of biomass and the purification of dye wastewater are urgent problems.The exceptional properties of MOFs, coupled with the superior attributes of the cellulose aerogel matrix, contribute to this high potential. This study explores the absorption properties of anionic dyes using a composite material comprising MOF-5 and cellulose aerogel. MOF-5 is renowned for its exceptional surface area and porosity, while cellulose aerogel provides structural support. The selection of anionic dyes indicates a deliberate targeting of negatively charged dyes, aiming to efficiently remove them from wastewater. The researchers conducted characterizations of the composite material and analyzed the adsorption mechanism to gain insights into the interactions between the composite and anionic dyes. The anticipated benefits of employing the MOF-5/cellulose aerogel composite include enhanced adsorption capacity, improved stability, and suitability for large-scale applications.

Also, we present a straightforward and innovative approach for crafting structured MOFs using pampas. Aerogels were synthesized through a basic sol–gel technique, succeeded by freeze-drying. We examined the capacity of these MOF-5/aerogel cellulose composite to absorb organic dyes.

## Materials and method

### Materials and instrumentation

All plant experiments and protocols adhered to relevant institutional, national, and international guidelines and legislation. The experimental procedure utilized pampas grass as the raw material, which was obtained through purchase. All chemicals employed in the experiment, including zinc nitrate hexahydrate, terephthalic acid, N,N-dimethylformamide, triethylamine, sodium hydroxide, sodium hypochlorite, nitric acid, Acid Blue, and deionized water, were commercially sourced and met the standards for analytical reagent grade.

### Preparation of MOF-5/cellulose aerogel composite

#### Extraction of cellulose

Initially, 5 g of pampas were meticulously weighed and combined with 1 M sodium hydroxide solution. The substance obtained from this step was then mixed in a solution of ethanol and nitric acid (ratio 5:1) and stirred for 180 min at a temperature ranging from 65 to 70 °C. Finally, the extracted cellulose was thoroughly washed multiple times using a sodium hypochlorite solution.

#### Synthesis of aerogel

One mole of cellulose extracted from pampas grass was mixed with 300 ml of distilled water and stirred for 1 h to disperse. After about an hour, 0.5 mol of methylene bisacrylamide were slowly added to the container until fully homogenized, and the mixture was stirred. The obtained mixture was then ultrasonicated for one hour at 25 °C to obtain a gel. The resulting gel was kept in the freezer for 12 h, followed by freeze-drying.

#### Synthesis of MOF-5

The synthesis of MOF-5 was carried out with reference to previous research and included innovations. Initially, a solution containing terephthalic acid, triethylamine, and DMF was prepared in specific proportions. Separately, a solution of sodium chloride and DMF was also prepared, and both solutions were combined at ambient temperature. Subsequently, a solution of zinc salt was gradually introduced into the initial mixture. The resulting solution is then transferred to an autoclave, a specialized reaction vessel designed to withstand high temperatures and pressures. The autoclave is heated to 383 K (equivalent to approximately 110 °C) for a duration of 24 h to facilitate the reaction. Subsequently, the synthesized MOF-5 undergo centrifugation and are washed with dimethylformamide (DMF).

#### Synthesis of MOF-5/Cellulose aerogel composite

**Figure 2:**
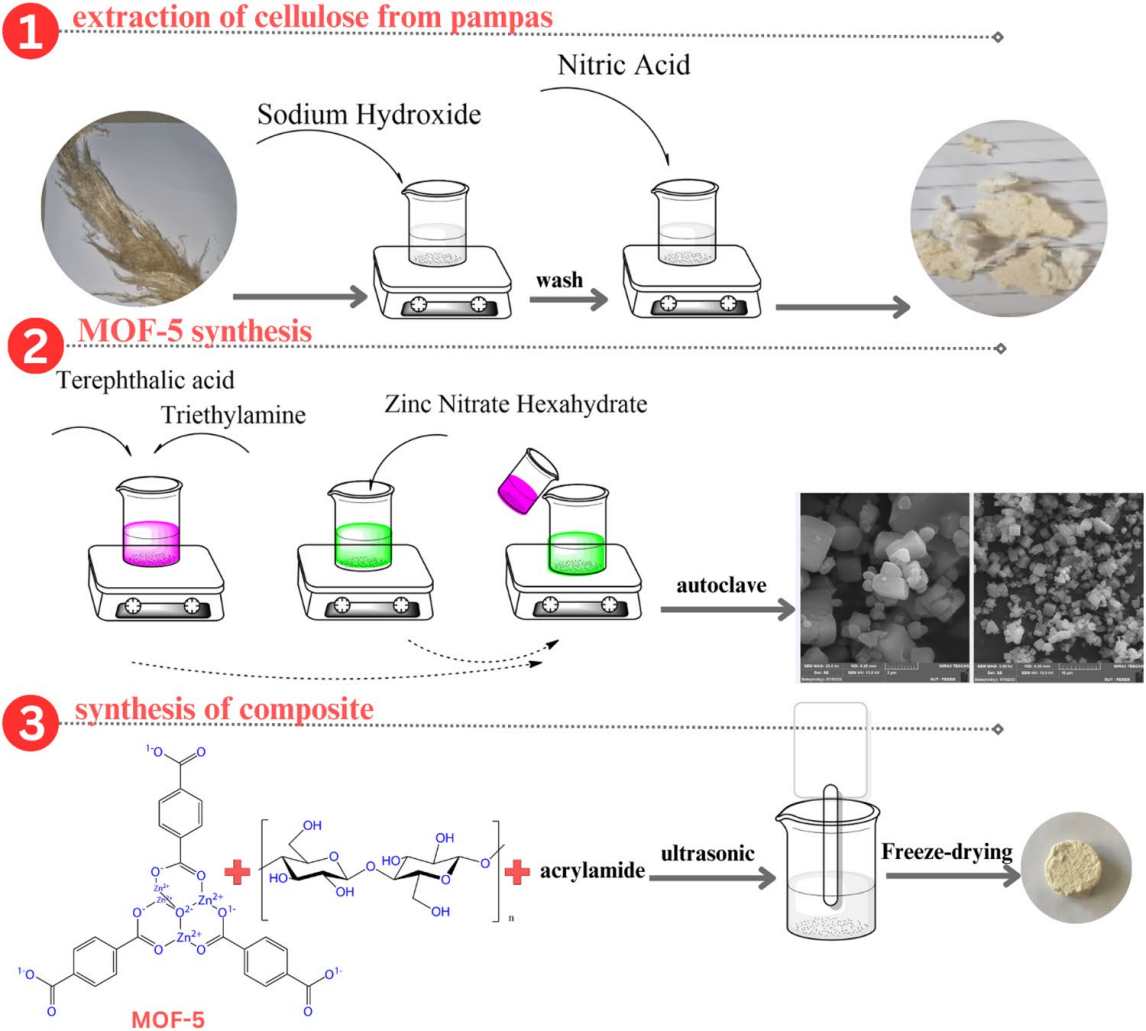
Production steps of MOF-5/cellulose aerogel composites produced from pampas grass.

According to the outlined procedure, the initial step involves blending acrylamide with cellulose extract in a water-based solvent. Following this, a MOF is introduced into the mixture. To optimize the manufacturing method, different amounts of MOF-5 were added to the aerogel, and its performance in removing the coloring agent was evaluated (the results are mentioned in Table 1). The mixture was then carefully mixed and sonicated at 90 °C for one hour. Finally, the material underwent freeze-drying. Figure 2 offers a visual depiction of the process for producing MOF-5/cellulose aerogel composites from pampas grass.

**Table 1:** Optimizing the production method of MOF-5/Cellulose aerogel composite.

Entry	MOF	Adsorption (%)
Composite 1	0.01	50
Composite 2	0.1	96

### Analyses and instrumentation

The MOF-5/Cellulose aerogel composite underwent surface morphology analysis using scanning electron microscopy (SEM) (Hitachi Su3500 model from Japan). This technique enables detailed examination of the sample’s topography, texture, and composition. SEM photographs were captured at room temperature via a scanning electron microscope, with the appropriate magnification selected to fulfil specific analysis requirements. SEM images were obtained at various magnifications to capture both fine details and an overall view of the sample. These images provide crucial insights into the surface structure of the composite, revealing morphological features such as cracks, pores, and roughness. Understanding these characteristics aids researchers in determining material properties, including porosity, surface area, and potential applications. SEM analysis facilitates visualization and examination of the composite material’s microstructure, offering valuable information for further characterization and optimization.

For determining absorption amounts, we utilized the Shimadzu 1800 model UV spectrophotometer from Japan. This method accurately quantifies the absorption or transmission of ultraviolet and visible light by the sample, offering insight into the effectiveness of the dye removal process.

X-ray diffraction (XRD) analysis was conducted using the XMD300 model from Switzerland. By passing X-rays through a crystal and measuring the resulting diffraction pattern, valuable information about the crystal’s atomic arrangement can be obtained. Analyzing the diffraction pattern enables researchers to determine the crystal’s lattice parameters, unit cell dimensions, and the positions of atoms within the crystal lattice. These details provide insights into the crystal’s structure, facilitating material identification and property investigation.

Functional group analysis was performed using Fourier-transform infrared (FTIR) spectroscopy, employing the Perkin Elmer model from the USA. The compound also underwent BET analysis utilizing N2 adsorption. The adsorption–desorption isotherm of the synthesized compound was then examined.

### Adsorption experiments

Adsorption tests were conducted to investigate the absorption characteristics of the cellulose aerogel composite with MOF-5. The experimental procedure involved synthesizing and characterizing the MOF-5/Cellulose aerogel composite. Subsequently, a comparative analysis was performed to assess their adsorption capacity and kinetic modelling, specifically for removing acid blue (an anionic dye). This method enabled the accurate determination of the rate at which dyes were adsorbed onto the porous materials. Following the kinetic analysis, a comprehensive parametric evaluation was conducted with utmost precision.

Throughout the experiments, a range of parameters were tested to analyze their influence on the dye adsorption process. These parameters included pH levels ranging from 3 to 10, initial dye concentrations ranging from 5 to 100 mg/L, mixing times ranging from 2 to 180 min, and adsorbent doses ranging from 0.1 to 1 g/L. Multiple adsorption experiments were carried out on the MOF-5/Cellulose aerogel composite for dye removal. Upon completion of the adsorption period, centrifugation was employed to separate the materials and dye solution, ensuring the isolation of materials from the liquid phase. These experiments yielded valuable insights into the potential applications of these materials in environmental contexts.

The Shimadzu UV-1800 spectrophotometric method was utilized to determine the color concentration in the liquid phase. In each experimental step, the percentage of dye removal (μ%) was calculated by subtracting the dye concentration after adsorption from the dye concentration before adsorption (Eq. 1). This calculation facilitated the quantification of adsorption performance and evaluation of the extent of dye removal from the porous material. The dye removal efficiency (μ%) for each experimental run was calculated by taking the difference between the dye concentration after adsorption (C, measured in mg/L) and the dye concentration before adsorption, according to Eq. (1). This calculation allowed researchers to quantify the effectiveness of the adsorption process and assess the extent of dye removal achieved by the porous materials.

1 $$\mu \left( \% \right) = \frac{{C_{0} - C}}{{C_{0} }} \times 100$$

The capacity of adsorbents at any given time (qt, mg/g) can be calculated using the following formula, considering the mass of adsorbents in the solution (m, g), the volume of the solution (V, L), initial dye concentration (C_0_, mg/L), and dye concentration at that specific time (C_t_, mg/g) in (Eq. 2):

The formula (Eq. 2) for calculating the adsorbent capacity at a specific time (qt, mg/g), considering the adsorbent amount in the solution (m, g), is as follows:

2 $$q_{t} = \left( {C_{0} - C_{t} } \right) \times \left( \frac{V}{m} \right)$$

### Study of reusability of adsorbent materials

The MOF-5/cellulose aerogel composite material employed for dye removal underwent washing with ethanol and was subsequently reused in further tests to evaluate its efficacy.

## Results and discussion

Metal–organic frameworks (MOFs) exhibit versatility in creating various structures with distinct properties, stemming from differences in metal ions and organic ligands^28–30^. Organic bonds within MOF structures play a pivotal role in determining their characteristics, including thermal and aqueous stability, as well as absorption performance. The adsorption and pollutant removal capabilities of MOFs are comparable to those of other adsorbents. Notably, MOF-5 stands out as a significant organometallic structure^31^, characterized by its three-dimensional configuration comprising terephthalic acid ligands and a Zn_4_O (Zn_4_O(TPA)_3_) framework^32^.

Cellulose, being a natural polymer^33–44^, exhibits properties influenced by factors such as chain length (carbon number), size, and thermal stability. Additionally, these properties vary based on the plant species and extraction processes utilized^45,46^. The extracted cellulose serves as a precursor for aerogel production. The choice of plant source for cellulose extraction significantly impacts the structure and performance of resulting cellulose aerogels^47,48^, which are valued for their biocompatibility and biodegradability.

A composite material incorporating MOF-5 and cellulose aerogel was synthesized using pampas plants. MOF-5’s exceptional surface area and porosity, combined with cellulose aerogel’s structural support, enhance dye absorption. The selection of anionic dyes indicates a deliberate targeting of negatively charged dyes, aligning with the composite’s design to efficiently eliminate such contaminants from wastewater. Leveraging the innate structure and high cellulose content of pampas plants minimizes environmental impact in cellulose aerogel production.

Results suggest that the MOF-5/cellulose aerogel composite effectively removes anionic dyes. The composite’s adsorption performance was evaluated under varying contact times, dye concentrations, and pH levels. Utilizing the MOF-5/cellulose aerogel composite offers several anticipated advantages, including increased adsorption capacity, enhanced stability, and suitability for large-scale applications.

### Characterization of MOF-5/cellulose aerogel composite

#### Surface morphology study

**Figure 3:**
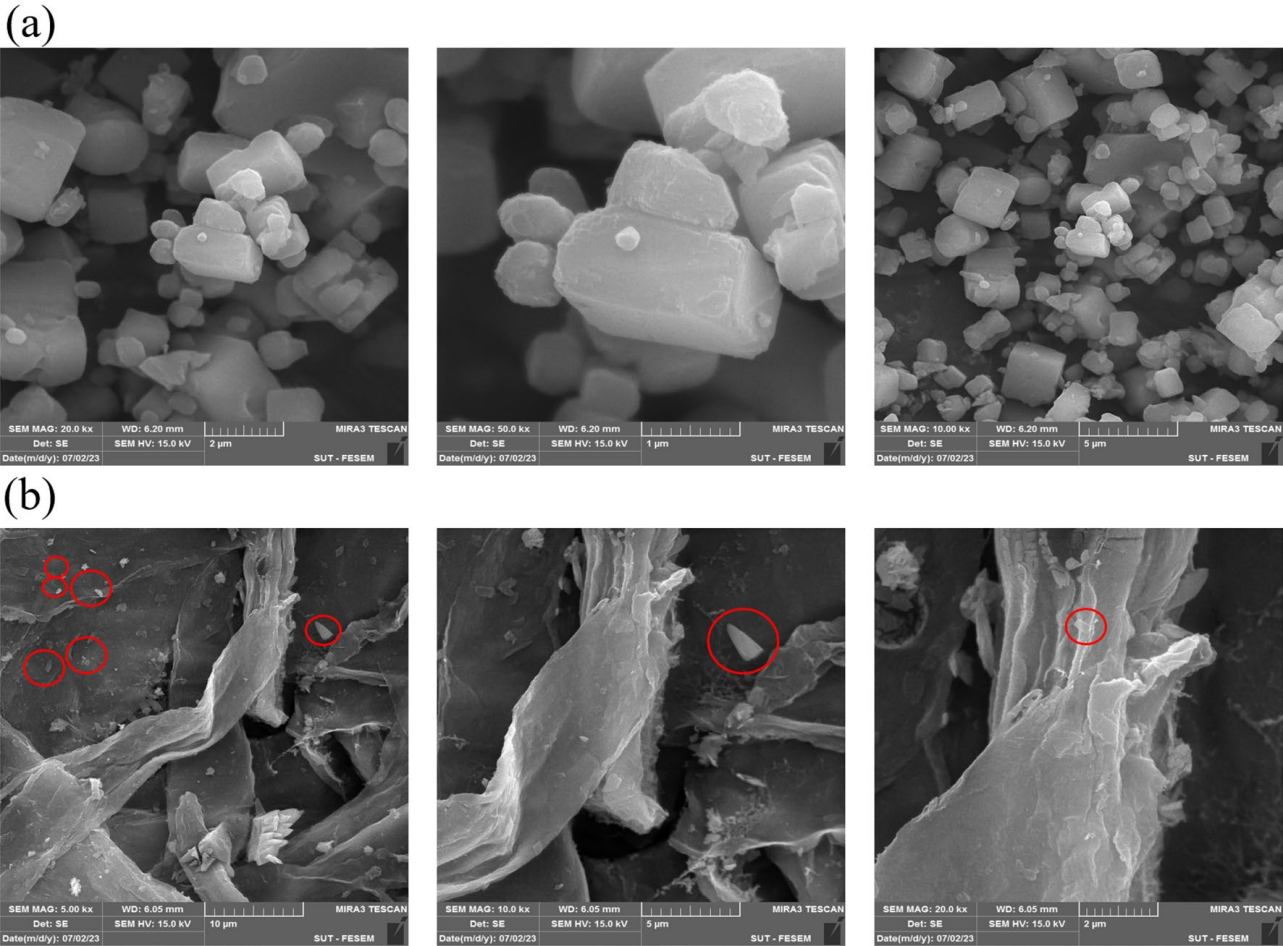
(**a**) SEM images of MOF-5 particles and (**b**) MOF-5/cellulose aerogel.

Scanning electron microscopy (SEM) was conducted on the fabricated composites to analyze their surface morphology, size, and shape. As depicted in Fig. 3a,b, SEM images of the MOF at different magnifications reveal a cubic structure with crystal sizes ranging from 80 to 150 nm. This structure aligns with previous findings in the literature concerning synthesized MOFs. Furthermore, Fig. 3b shows an SEM image of the MOF-5/cellulose aerogel composite, showcasing a notable morphological transformation possibly attributed to the chemical bonding of aerogels by MBA. The coordination of MBA with free electron pairs on nitrogen atoms alters the MOF morphology, facilitating the integration of MOF molecules with the aerogel solution and creating additional free space. The SEM images (Fig. 3a) clearly depict cubic crystals of almost uniform size, indicating successful MOF formation. Additionally, the SEM image of the MOF-5/cellulose aerogel composite (Fig. 3b) reveals a fibril-aggregated pattern of cellulose aerogel and MOF-5 crystals around fibers, affirming successful synthesis. This characterization confirms the composite’s suitability for intended applications, such as adsorption-based processes or environmental remediation.

#### XRD

**Figure 4:**
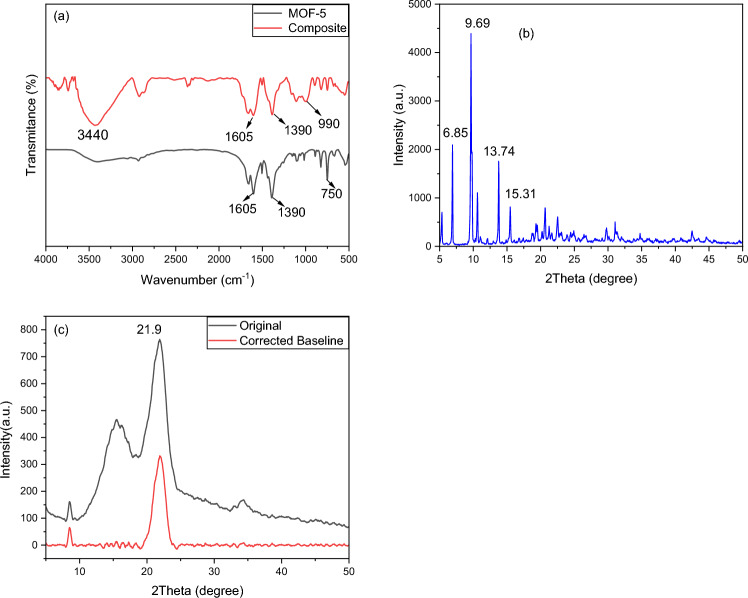
(**a**) IR spectrum for MOF and Comparison of IR spectrum of MOF-5/cellulose aerogel composite (**b**) XRD patterns of MOF-5 (**c**) XRD patterns of MOF-5/cellulose aerogel composite.

In Fig. 4b of the study, XRD patterns for the MOF-5 are presented. The XRD spectrum for MOF-5 exhibits characteristic peaks at 6.85 degrees, 9.69 degrees, 13.74 degrees, and 15.31 degrees. These peaks align with the pattern specified in the literature, indicating the presence of MOF-5 with its expected crystal structure (as reported in reference^49^ 46). Furthermore, the XRD pattern for the MOF-5/cellulose aerogel composite in Fig. 4c displays a prominent peak in the 2θ range between 20 and 30 degrees, characteristic of cellulose^50,51^.

#### Fourier transform infrared spectroscopic analysis (FTIR)

The FTIR spectrum for MOF-5 is depicted in Fig. 4a. Several distinct peaks are identifiable, including asymmetric and symmetric stretching of C–C at 1390 cm^−1^, C=C stretching at 1605 cm^−1^, and metal–oxygen bond (Zn–O) represented by notable bands at 750 cm^−1^, serving as a chemical fingerprint of MOF-5^52^. The FTIR spectra also provide insights into the MOF-5/cellulose aerogel composite material. The presence of hydroxyl groups (–OH) is indicated by a peak at 3400 cm^−1^. Additionally, peak corresponding to C–O group is observed at 1080 cm^−1^, respectively, within the MOF-5/cellulose aerogel composite structure. Moreover, when considering MOF-5 formed encasing the fibers, the prominent peaks at approximately 1390 and 1600 cm^−1^ indicate the presence of the organic linker, specifically terephthalic acid in the case of MOF-5^53^.

#### BET

**Figure 5:**
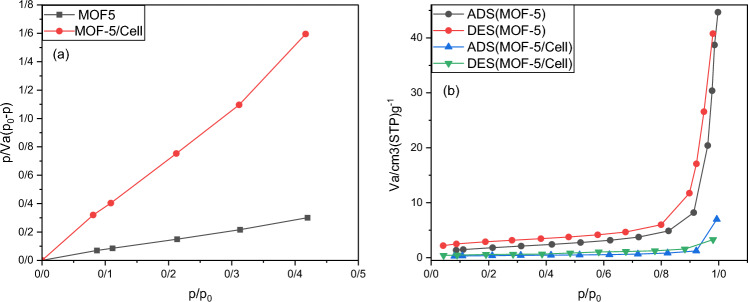
(**a**) Nitrogen adsorption–desorption isotherms of MOf-5 and MOF-5/cellulose aerogel composite (**b**) BET surface area plot.

The differences in the porous properties of MOF-5 and MOF-5/cellulose aerogel composites were confirmed through N_2_ adsorption tests. The results of these tests are represented in a graph shown in Fig. 5, which displays the N_2_ adsorption–desorption isotherm (Fig. 5a) and BET surface area plot (Fig. 5b). According to Brunauer’s classification of five types of adsorption isotherms, it is suggested that the adsorption isotherms of MOF-5 and MOF-5/cellulose aerogel composites likely fall under type IV isotherms, with hysteresis loops belonging to H3 and H1 categories. The isotherm is described as being convex at low pressure, indicating a strong interaction between the adsorbate and the sorbent. According to the International Union of Pure and Applied Chemistry (IUPAC), MOF-5 and MOF-5/cellulose aerogel composites are classified as mesoporous, given that the width of the mesopores ranges between 2 and 50 nm.

Additionally, the specific surface area, pore size, and pore volume are calculated and the results are shown in Table 2.

**Table 2:** BET surface area, pore size, pore volume of MOF-5, MOF-5/cellulose aerogel composite.

Sample	BET surface area (m^2^ g^−1^)	Pore volume (cm^3^g^−1^)	Pore size (nm)
MOF-5	6.4197	0.0634	39.497
MOF/Cell	1.2637	0.2903	32.827

#### UV–Vis

**Figure 6:**
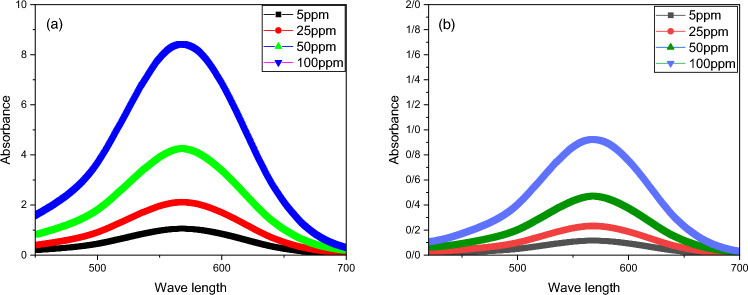
Illustrates the UV–Vis spectra of solutions before (**a**) and after (**b**) exposure to MOF-5 cellulose aerogel composite. The exposure duration was 180 min.

To assess the adsorption ability of the MOF-5/cellulose aerogel composite, acid blue dye was selected from numerous aqueous pollutants. In the experiment, a small piece of the MOF-5/cellulose aerogel composite was immersed in solutions containing acid blue dye. Over time, the color of the solution gradually diminished, eventually becoming colorless. Concurrently, the UV–Vis absorption peak at 610 nm exhibited a significant reduction, as depicted in Fig. 6. Interestingly, the color of the MOF-5/cellulose aerogel composite transformed from white to blue. This change indicates that the MOF-5/cellulose aerogel composite effectively adsorbed the acid blue molecules. The significant size of acid blue molecules, exceeding 200 nm (larger than the 80 nm pore diameter of the MOF), prevents their entry into the MOF’s pores, resulting in surface adsorption instead. MOF-5 may contain metal sites capable of forming coordination bonds with acid blue molecules, while aerogels may provide surface functional groups (such as hydroxyl or amino groups) capable of hydrogen bonding or electrostatic interactions with the dye molecules. These interactions help trap the acid blue molecules within the composite. The combination of MOF-5 and aerogels in a composite can enhance the overall adsorption capacity and efficiency compared to either material alone. This could be due to increased surface area, improved pore accessibility, or enhanced chemical interactions between the composite and acid blue molecules.

To determine the maximum absorption capacity of aqueous acid for our composite, we conducted absorption isotherm studies at various initial concentrations while maintaining a constant temperature of 25 °C. As depicted in Table 3, the equilibrium adsorption data closely adheres to the Langmuir model, revealing a maximum adsorption capacity of up to 60 mg/g.

### Zeta potential and point-zero charge measurements

The surface charge properties of the prepared composite material, MOF-5/cellulose aerogel, were examined through zeta potential measurements and determination of pHpzc. The MOF-5/cellulose aerogel composite exhibited a positive surface charge, with an average zeta potential of 35.34 mV. The pHpzc value, representing the pH at which the surface charge is neutral, was determined to be 6.8 in water.

At pH values lower than pHpzc, the surface of the composite develops a positive charge, resulting in increased absorption. Consequently, the absorption of the anionic dye was notably higher in acidic pH conditions, where the positively charged surface facilitated electrostatic attraction, leading to a faster absorption process and enhanced overall absorption capacity.

#### The effect of pH on absorption

**Figure 7:**
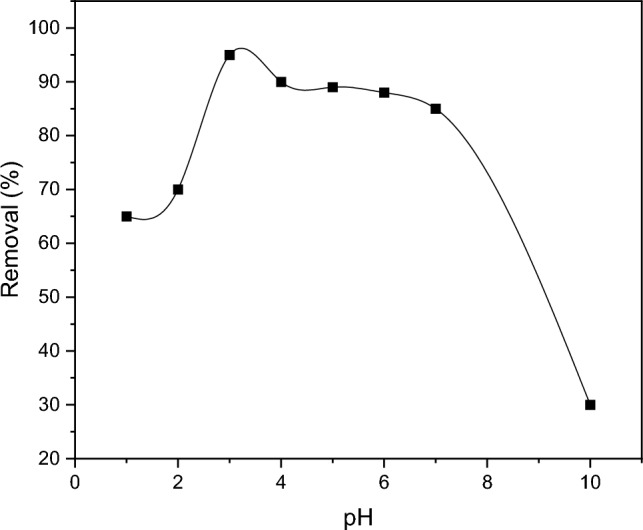
The effect of pH on the adsorption.

The impact of pH levels on dye removal by the MOF-5/cellulose aerogel composite is illustrated in Fig. 7. Specifically, for the absorption of the anionic pigment acid blue, the optimal pH level is acidic, particularly at pH 3.

### Absorbance study of MOF-5/cellulose aerogel composite

#### Absorption kinetics

The adsorption kinetics of MOF-5/cellulose aerogel composite was explained and analyzed by using different mathematical models in Eqs. (3 and 4). The pseudo-first-order kinetic model (Eq. 3) and the pseudo-second-order model (Eq. 4).

In this context, qt (mg/g) and qe (mg/g) represent the adsorption capacity at time t (minutes) and at equilibrium, respectively. k_1_ and k_2_ denote the rate constants for the pseudo-first-order (min^−1^) and pseudo-second-order adsorption kinetics (g mg^−1^ min^−1^)

3 $$\log \left( {{\text{q}}_{{\text{e}}} - {\text{q}}_{{\text{t}}} } \right) = {\text{logq}}_{{\text{e}}} - \frac{{{\text{K}}_{1} {\text{ t}}}}{2.303}$$

4 $$\frac{{\text{t}}}{{{\text{q}}_{{\text{t}}} }} = { }\frac{1}{{{\text{K}}_{2} {\text{q}}_{{\text{e}}}^{2} }} + \frac{1}{{{\text{q}}_{{\text{e}}} }}{\text{t}}$$

**Figure 8:**
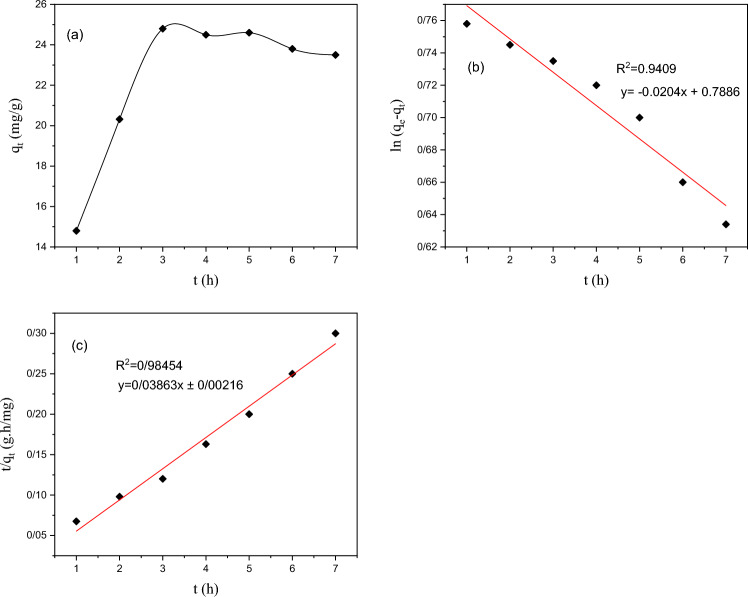
(**a**) The Effect of Contact Time on Adsorption Capacity (**b**) Pseudo-First-Order Kinetic Model (**c**) Pseudo-Second-Order Kinetic Model of Acid Blue Dye and MOF-5/Cellulose Aerogel Absorbent.

Mathematical models were employed to calculate the correlation coefficient (R^2^) and release rate constant (K) values through regression analysis. These values provide valuable insights into release kinetics and mechanisms. The results depicted in Fig. 8 indicate that the pseudo-second-order kinetic model exhibits the highest regression coefficient, indicating its superior accuracy.

The pseudo-second-order model suggests that the rate of absorption is directly proportional to the square of the difference between the amount of absorption in the equilibrium state and the amount of absorption at any given time, which is utilized to analyze absorption processes. In this model, the rate-controlling step is determined based on the chemical adsorption of the adsorbate onto the surface of the adsorbent.

#### Adsorption isotherm

Adsorption isotherm models are pivotal in evaluating absorption progress and investigating absorption mechanisms. In this study, the equilibrium data of the MOF-5/cellulose aerogel composite were analyzed using the Langmuir, Freundlich, and Temkin models, as described by Eqs. (5–7). The application of these isotherm models in adsorption treatment was scrutinized through the assessment of the correlation coefficient (R^2^)66,65.

5 $$\frac{Ce \, }{{qe}} \, = \, \frac{1 \, }{{q_{m} \cdot K_{L} }} \, + \, \frac{{C_{e} }}{{ \, q_{m} }}$$

6 $$\frac{X}{m} \, = \, Kp^{{\frac{{^{1} }}{n}}}$$

7 $$q = \, B \, \ln \, \left( {A \, C} \right)$$

The Langmuir isotherm model, grounded in the concept that the adsorption process occurs at homogeneous sites on the adsorbent surface, was employed. According to this model, once the dye fills the adsorbent surface, no further adsorption can occur on that surface. This suggests that the adsorption process inherently follows a monolayer mechanism. The linear form of the Langmuir isotherm model is expressed by Eq. (5) ^54^.

The Freundlich isotherm describes the physico-chemical process in which the adsorbate is transferred from the solution phase into the porous adsorbent, where the adsorption process takes place. The mathematical representation of the Freundlich isotherm model is expressed by Eq ^55^. The Temkin isotherm model posits that the adsorption heat of all molecules diminishes linearly as the coverage of the adsorbent surface increases. It assumes that adsorption is marked by a consistent distribution of binding energies, reaching a maximum binding energy. The mathematical expression for the Temkin isotherm model is then given by Eq. (7)

The results obtained for all models are presented in Table 3. The correlation coefficient (R^2^) value for the Langmuir model is 0.9753, which is higher than the values calculated for the Freundlich and Temkin isotherms. Therefore, it appears that the Langmuir isotherm better describes the adsorption of acid blue dye by the MOF-5/cellulose aerogel composite.

**Table 3:** Adsorption isotherm data for MOF-5/cellulose aerogel composite.

Isotherm	Parameters	MOF-5/cellulose aerogel composite
Langmuir	b	0.49
	q_e_	60
	R^2^	0.9753
Freundlich	K_f (mg/g)_	25.58
	n	6.16
	R^2^	0.9514
Temkin	B	117.81
	q	18.8
	R^2^	0.7841

q_m_ represents the maximum adsorption capacity (also known as the Langmuir adsorption capacity), indicating the maximum amount of adsorbate that can be adsorbed per unit mass of adsorbent at monolayer coverage. According to the obtained results, the maximum adsorption capacity for the manufactured composite is 60 mg/g.

The Langmuir isotherm model, based on the assumption that adsorption transpires at uniform sites on the adsorbent surface, was utilized. According to this model, once the surface of the adsorbent is completely covered by the color, further adsorption ceases on that surface^54^.

### Regeneration study of MOF-5/cellulose aerogel composite

The recyclability of adsorbents is a crucial aspect to consider. Hence, we examined the recycling performance of the MOF-5/cellulose aerogel composite. The results demonstrated that it maintained its excellent adsorption performance and capacity even after multiple recycling cycles.

**Figure 9:**
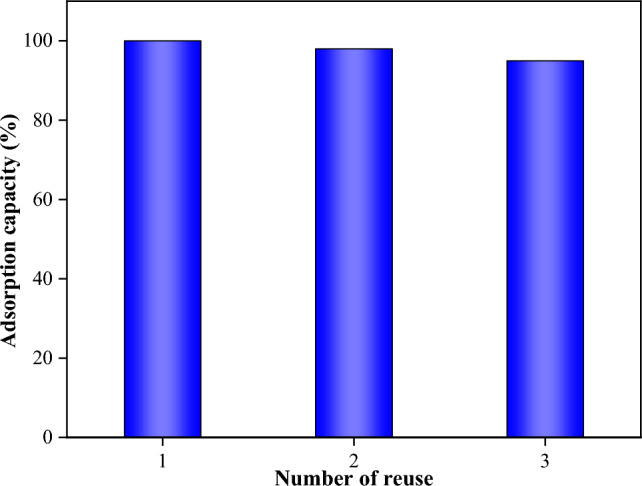
The result of composite recovery and reuse.

Figure 9 illustrates the results of the reuse of the MOF-5/cellulose aerogel composite over three cycles. For each reuse, the composite material was washed with diluted ethanol as a washing agent to remove aqueous acid. After three reuses, the adsorption capacity of the MOF-5/cellulose aerogel composite decreased by only 5%.

### Comparison of other absorbents

The results of the maximum absorption capacity of the MOF-5/cellulose aerogel composite for dye adsorption are presented in Table 4. The obtained results demonstrate the good adsorption capacity of the MOF composite compared to other adsorbents.

**Table 4:** Previous studies on maximum absorbed values.

Adsorbent	qm (mg/g)	Ref
MOF-199	15.28	^56^
Fe_3_O_4_@MIL-100 (Fe)	49.41	^57^
Activated carbon	46	^58^
MOF-5/cellulose aerogel composite	60	This work

## Conclusion

A novel composite adsorbent comprising MOF-5 and cellulose aerogel was employed for the absorption of a specific dye type, acid blue (anionic). Under neutral conditions (pH 7.8) and a temperature of 45 degrees Celsius, with a concentration of 50 mg of dye and 0.1 g of absorbent over a duration of 180 min, 76.58% of the dye was removed. Conversely, under acidic conditions (pH 3) at ambient temperature, 96% of the dye was removed in less than 60 min. These results highlight the significant influence of pH on the removal process.

Furthermore, the absorbent material exhibited reusability, presenting an eco-friendly solution for dye removal. The cellulose aerogel MOF-5 adsorbent demonstrated consistent performance over several consecutive cycles. Thus, this adsorbent combination proves to be an efficient solution for the removal of acid blue dye from water.

## Data Availability

All data generated or analyzed data for the experimental part of this study are available from the corresponding author (Majid Hosseinzadeh), upon reasonable request.

## References

[1] Maleki, H. & Hüsing, N. Current status, opportunities and challenges in catalytic and photocatalytic applications of aerogels: Environmental protection aspects. *Appl. Catal. B* **221**, 530–555 (2018). 10.1016/j.apcatb.2017.08.012

[2] Hooriabad Saboor, F., Nasirpour, N., Shahsavari, S. & Kazemian, H. The effectiveness of MOFs for the removal of pharmaceuticals from aquatic environments: A review focused on antibiotics removal. *Chem. Asian J.* **17**, e202101105 (2022). 34941022 10.1002/asia.202101105

[3] Adeleye, A. S. *et al.* Engineered nanomaterials for water treatment and remediation: Costs, benefits, and applicability. *Chem. Eng. J.* **286**, 640–662 (2016). 10.1016/j.cej.2015.10.105

[4] Kadhom, M., Albayati, N., Alalwan, H. & Al-Furaiji, M. Removal of dyes by agricultural waste. *Sustain. Chem. Pharm.* **16**, 100259 (2020). 10.1016/j.scp.2020.100259

[5] Pathania, D., Sharma, S. & Singh, P. Removal of methylene blue by adsorption onto activated carbon developed from Ficus carica bast. *Arab. J. Chem.* **10**, S1445–S1451 (2017). 10.1016/j.arabjc.2013.04.021

[6] De Gisi, S., Lofrano, G., Grassi, M. & Notarnicola, M. Characteristics and adsorption capacities of low-cost sorbents for wastewater treatment: A review. *Sustain. Mater. Technol.* **9**, 10–40 (2016).

[7] Kyzas, G. Z. & Kostoglou, M. Green adsorbents for wastewaters: A critical review. *Materials* **7**, 333–364 (2014). 28788460 10.3390/ma7010333 PMC5453162

[8] Abbasi, Z., Cseri, L., Zhang, X., Ladewig, B. P. & Wang, H. *In sustainable nanoscale engineering* 163–194 (Elsevier, Amsterdam, 2020).

[9] Wen, M. *et al.* Metal–organic framework-based nanomaterials for adsorption and photocatalytic degradation of gaseous pollutants: Recent progress and challenges. *Environ. Sci.: Nano* **6**, 1006–1025 (2019).

[10] Giannakoudakis, D. A. *et al.* Ultrasound-activated TiO2/GO-based bifunctional photoreactive adsorbents for detoxification of chemical warfare agent surrogate vapors. *Chem. Eng. J.* **395**, 125099 (2020). 10.1016/j.cej.2020.125099

[11] Ghadiri, S. K. *et al.* Adsorption of nitrate onto anionic bio-graphene nanosheet from aqueous solutions: Isotherm and kinetic study. *J. Mol. Liq.* **242**, 1111–1117 (2017). 10.1016/j.molliq.2017.06.122

[12] Luan, T. B. Dye adsorption on UiO-66: The importance of electrostatic attraction mechanism. *J. Water Chem. Technol.* **42**, 441–449 (2020). 10.3103/S1063455X20060107

[13] Li, S. & Huo, F. Metal–organic framework composites: From fundamentals to applications. *Nanoscale* **7**, 7482–7501 (2015). 25871946 10.1039/C5NR00518C

[14] Xia, Z. *et al.* Processing and valorization of cellulose, lignin and lignocellulose using ionic liquids. *J. Bioresour. Bioprod.* **5**, 79–95 (2020). 10.1016/j.jobab.2020.04.001

[15] Zhang, X. F. *et al.* Inorganic salts induce thermally reversible and anti-freezing cellulose hydrogels. *Angewandte Chem. Int. Edition* **58**, 7366–7370 (2019). 10.1002/anie.201902578 30938928

[16] Mo, L., Pang, H., Tan, Y., Zhang, S. & Li, J. 3D multi-wall perforated nanocellulose-based polyethylenimine aerogels for ultrahigh efficient and reversible removal of Cu (II) ions from water. *Chem. Eng. J.* **378**, 122157 (2019). 10.1016/j.cej.2019.122157

[17] Shang, Q., Hu, Y., Liu, C., Yang, X. & Zhou, Y. Fabrication of superhydrophobic cellulose composite aerogels for oil/water separation. *J Forest. Eng.* **4**, 86–92 (2019).

[18] Zhang, X.-F. *et al.* Highly transparent graphene oxide/cellulose composite film bearing ultraviolet shielding property. *Int. J. Biol. Macromol.* **145**, 663–667 (2020). 31891698 10.1016/j.ijbiomac.2019.12.241

[19] Chen, S. *et al.* Robust three-dimensional g-C3N4@ cellulose aerogel enhanced by cross-linked polyester fibers for simultaneous removal of hexavalent chromium and antibiotics. *Chem. Eng. J.* **359**, 119–129 (2019). 10.1016/j.cej.2018.11.110

[20] Yadav, C. *et al.* Plant-based nanocellulose: A review of routine and recent preparation methods with current progress in its applications as rheology modifier and 3D bioprinting. *Int. J. Biol. Macromol.* **166**, 1586–1616 (2021). 33186649 10.1016/j.ijbiomac.2020.11.038

[21] Narwade, V. N., Khairnar, R. S. & Kokol, V. In-situ synthesised hydroxyapatite-loaded films based on cellulose nanofibrils for phenol removal from wastewater. *Cellulose* **24**, 4911–4925 (2017). 10.1007/s10570-017-1435-2

[22] Pottathara, Y. B., Narwade, V. N., Bogle, K. A. & Kokol, V. TEMPO-oxidized cellulose nanofibrils–graphene oxide composite films with improved dye adsorption properties. *Polym. Bull.* **77**, 6175–6189 (2020). 10.1007/s00289-019-03077-3

[23] Zhu, W. *et al.* Amino-functionalized nanocellulose aerogels for the superior adsorption of CO2 and separation of CO2/CH4 mixture gas. *Carbohydr. Polym.* **323**, 121393 (2024). 37940286 10.1016/j.carbpol.2023.121393

[24] Zhu, W. *et al.* Fibrous cellulose nanoarchitectonics on N-doped Carbon-based Metal-Free catalytic nanofilter for highly efficient advanced oxidation process. *Chem. Eng. J.* **460**, 141593 (2023). 10.1016/j.cej.2023.141593

[25] Wang, Z. *et al.* Lightweight UiO-66/cellulose aerogels constructed through self-crosslinking strategy for adsorption applications. *Chem. Eng. J.* **371**, 138–144 (2019). 10.1016/j.cej.2019.04.022

[26] Liu, R. *et al.* Effective and selective adsorption of phosphate from aqueous solution via trivalent-metals-based amino-MIL-101 MOFs. *Chem. Eng. J.* **357**, 159–168 (2019). 10.1016/j.cej.2018.09.122

[27] Wang, Y. *et al.* The advances of polysaccharide-based aerogels: Preparation and potential application. *Carbohydr. Polym.* **226**, 115242 (2019). 31582065 10.1016/j.carbpol.2019.115242

[28] Haldar, D., Duarah, P. & Purkait, M. K. MOFs for the treatment of arsenic, fluoride and iron contaminated drinking water: A review. *Chemosphere* **251**, 126388 (2020). 32443223 10.1016/j.chemosphere.2020.126388

[29] Gautam, S. *et al.* Metal oxides and metal organic frameworks for the photocatalytic degradation: A review. *J. Environ. Chem. Eng.* **8**, 103726 (2020). 10.1016/j.jece.2020.103726

[30] Jiang, D. *et al.* The application of different typological and structural MOFs-based materials for the dyes adsorption. *Coord. Chem. Rev.* **380**, 471–483 (2019). 10.1016/j.ccr.2018.11.002

[31] Burgaz, E., Erciyes, A., Andac, M. & Andac, O. Synthesis and characterization of nano-sized metal organic framework-5 (MOF-5) by using consecutive combination of ultrasound and microwave irradiation methods. *Inorgan. Chim. Acta* **485**, 118–124 (2019). 10.1016/j.ica.2018.10.014

[32] Xu, S. *et al.* Metal–organic framework-5 as a novel phosphorescent probe for the highly selective and sensitive detection of Pb (II) in mussels. *Sens. Actuators B: Chem.* **308**, 127733 (2020). 10.1016/j.snb.2020.127733

[33] Moon, R. J., Martini, A., Nairn, J., Simonsen, J. & Youngblood, J. Cellulose nanomaterials review: Structure, properties and nanocomposites. *Chem. Soc. Rev.* **40**, 3941–3994 (2011). 21566801 10.1039/c0cs00108b

[34] Aegerter, M. A., Leventis, N. & Koebel, M. M. *Advances in sol-gel derived materials and technologies* (Aerogels Handbook Springer, New York, 2011).

[35] Zhao, S., Malfait, W. J., Guerrero-Alburquerque, N., Koebel, M. M. & Nyström, G. Biopolymer aerogels and foams: Chemistry, properties, and applications. *Angewandte Chem. Int. Edition* **57**, 7580–7608 (2018). 10.1002/anie.201709014 29316086

[36] Liu, H., Geng, B., Chen, Y. & Wang, H. Review on the aerogel-type oil sorbents derived from nanocellulose. *ACS Sustain. Chem. Eng.* **5**, 49–66 (2017). 10.1021/acssuschemeng.6b02301

[37] De France, K. J., Hoare, T. & Cranston, E. D. Review of hydrogels and aerogels containing nanocellulose. *Chem. Mater.* **29**, 4609–4631 (2017). 10.1021/acs.chemmater.7b00531

[38] Fernandes, E. M., Pires, R. A., Mano, J. F. & Reis, R. L. Bionanocomposites from lignocellulosic resources: Properties, applications and future trends for their use in the biomedical field. *Progr. Polym. Sci.* **38**, 1415–1441 (2013). 10.1016/j.progpolymsci.2013.05.013

[39] Virtanen, T. *et al.* A physico-chemical characterisation of new raw materials for microcrystalline cellulose manufacturing. *Cellulose* **19**, 219–235 (2012). 10.1007/s10570-011-9636-6

[40] Reddy, N. & Yang, Y. Properties and potential applications of natural cellulose fibers from the bark of cotton stalks. *Bioresour. Technol.* **100**, 3563–3569 (2009). 19327987 10.1016/j.biortech.2009.02.047

[41] Wang, X., Li, H., Cao, Y. & Tang, Q. Cellulose extraction from wood chip in an ionic liquid 1-allyl-3-methylimidazolium chloride (AmimCl). *Bioresour. Technol.* **102**, 7959–7965 (2011). 21684735 10.1016/j.biortech.2011.05.064

[42] Cara, C., Ruiz, E., Ballesteros, I., Negro, M. J. & Castro, E. Enhanced enzymatic hydrolysis of olive tree wood by steam explosion and alkaline peroxide delignification. *Process Biochem.* **41**, 423–429 (2006). 10.1016/j.procbio.2005.07.007

[43] Abe, K. & Yano, H. Comparison of the characteristics of cellulose microfibril aggregates of wood, rice straw and potato tuber. *Cellulose* **16**, 1017–1023 (2009). 10.1007/s10570-009-9334-9

[44] Sun, J., Sun, X., Zhao, H. & Sun, R. Isolation and characterization of cellulose from sugarcane bagasse. *Polym. Degrad. Stab.* **84**, 331–339 (2004). 10.1016/j.polymdegradstab.2004.02.008

[45] Trache, D. *et al.* Microcrystalline cellulose: Isolation, characterization and bio-composites application—A review. *Int. J. Biol. Macromol.* **93**, 789–804 (2016). 27645920 10.1016/j.ijbiomac.2016.09.056

[46] Siqueira, G., Bras, J. & Dufresne, A. Cellulosic bionanocomposites: A review of preparation, properties and applications. *Polymers* **2**, 728–765 (2010). 10.3390/polym2040728

[47] Lee, S., Jeong, M.-J. & Kang, K.-Y. Preparation of cellulose aerogels as a nano-biomaterial from lignocellulosic biomass. *J. Korean Phys. Soc.* **67**, 738–741 (2015). 10.3938/jkps.67.738

[48] Jeong, M.-J., Lee, S., Kang, K.-Y. & Potthast, A. Changes in the structure of cellulose aerogels with depolymerization. *J. Korean Phys. Soc.* **67**, 742–745 (2015). 10.3938/jkps.67.742

[49] Yu, C.-X. *et al.* Highly efficient and selective removal of anionic dyes from aqueous solution by using a protonated metal-organic framework. *J. Alloys Compds.* **853**, 157383 (2021). 10.1016/j.jallcom.2020.157383

[50] Tian, S. *et al.* Highly efficient removal of both cationic and anionic dyes from wastewater with a water-stable and eco-friendly Fe-MOF via host-guest encapsulation. *J. Clean. Prod.* **239**, 117767 (2019). 10.1016/j.jclepro.2019.117767

[51] Yang, J.-M. *et al.* Adsorptive removal of organic dyes from aqueous solution by a Zr-based metal–organic framework: Effects of Ce (III) doping. *Dalton Trans,* **47**, 3913–3920 (2018). 29451582 10.1039/C8DT00217G

[52] Firoozi, M., Rafiee, Z. & Dashtian, K. New MOF/COF hybrid as a robust adsorbent for simultaneous removal of auramine O and rhodamine B dyes. *ACS Omega* **5**, 9420–9428 (2020). 32363294 10.1021/acsomega.0c00539 PMC7191862

[53] Song, J., Zhang, B., Jiang, T., Yang, G. & Han, B. Synthesis of cyclic carbonates and dimethyl carbonate using CO 2 as a building block catalyzed by MOF-5/KI and MOF-5/KI/K 2 CO 3. *Front. Chem. China* **6**, 21–30 (2011). 10.1007/s11458-011-0225-x

[54] Liang, Z., Marshall, M. & Chaffee, A. L. CO2 adsorption, selectivity and water tolerance of pillared-layer metal organic frameworks. *Microporous Mesoporous Mater.* **132**, 305–310 (2010). 10.1016/j.micromeso.2009.11.026

[55] Freundlich, H. Über die adsorption in lösungen. *Zeitschrift für physikalische Chemie* **57**, 385–470 (1907). 10.1515/zpch-1907-5723

[56] Shao, Y. *et al.* Magnetic responsive metal–organic frameworks nanosphere with core–shell structure for highly efficient removal of methylene blue. *Chem. Eng. J.* **283**, 1127–1136 (2016). 10.1016/j.cej.2015.08.051

[57] Zhang, C.-F. *et al.* A novel magnetic recyclable photocatalyst based on a core–shell metal–organic framework Fe 3 O 4@ MIL-100 (Fe) for the decolorization of methylene blue dye. *J. Mater. Chem. A* **1**, 14329–14334 (2013). 10.1039/c3ta13030d

[58] Liu, T. *et al.* Adsorption of methylene blue from aqueous solution by graphene. *Colloids Surf. B: Biointerfaces* **90**, 197–203 (2012). 22036471 10.1016/j.colsurfb.2011.10.019

