# Author Correction: Adsorbent based on MOF-5/cellulose aerogel composite for adsorption of organic dyes from wastewater

**DOI:** 10.1038/s41598-024-70735-6

**Published:** 2024-08-30

**Authors:** Mohammad Shiri, Majid Hosseinzadeh, Soudeh Shiri, Shahrzad Javanshir

**Affiliations:** 1 School of Civil Engineering, Iran University of Science and Technology, Narmak, Tehran, Iran; 2 Department of Organic Colorants, Institute of Color Science and Technology, Tehran, Iran; 3 Pharmaceutical and Heterocyclic Chemistry Research Laboratory, Department of Chemistry, Iran University of Science and Technology, Tehran, 16846-13114 Iran

Correction to: *Scientific Reports* 10.1038/s41598-024-65774-y, published online 07 July 2024

In the original version of this Article, incorrect References were included in the Reference list.

- Naderian, D., Noori, R., Heggy, E., Bateni, S.M., Bhattarai, R., Nohegar, A. & Shrama, S. A water quality database for global lakes. *Resour. Cons. Recy.* **202**,107401 (2024).
- Kim, H.I., Kim, D., Mahdian, M., Salamattalab, M.M. & Noori, R. Incorporation of water quality index models with machine learning-based techniques for real-time assessment of aquatic ecosystems. *Environ. Pollut.* **355**, 124242 (2024).
- Alizadeh, M., Noori, R., Omidvar, B., Nohegar, A. & Pistre, S. Human health risk of nitrate in groundwater of Tehran–Karaj plain Iran. *Sci. Rep.* **14**, 7830 (2024).
- Mahdian, M. et al*.* Modelling impacts of climate change and anthropogenic activities on inflows and sediment loads of wetlands: case study of the Anzali wetland. *Sci. Rep.* **13**, 5399 (2023).
- Noori, R., Ghiasi, B., Sheikhian, H. & Adamowski, J.F. Estimation of the dispersion coefficient in natural rivers using a granular computing model. *J. Hydraul. Eng.* **143**, 04017001 (2017).
- Noori R., Woolway, R.I., Saari, M., Pulkkanen, M. & Kløve, N. Six decades of thermal change in a pristine lake situated north of the Arctic Circle. *Water Resour. Res.* **58**, e2021WR031543 (2022).

As a result, in the Introduction,

“Water is vital for all forms of life, encompassing humans, animals, and plants. Despite its critical role, escalating pollution levels and water scarcity have emerged as significant global environmental challenges, erecting substantial barriers^6261^. Industrial activities, daily human practices, and agricultural operations stand out as prominent contributors to water pollution^63,64^.”

now reads:

“Water is vital for all forms of life, encompassing humans, animals, and plants. Despite its critical role, escalating pollution levels and water scarcity have emerged as significant global environmental challenges, erecting substantial barriers. Industrial activities, daily human practices, and agricultural operations stand out as prominent contributors to water pollution.”

The original Article has been corrected.

